# Evaluation of ultra-early and dose-dependent edema and ultrastructural changes in the myocyte during anti-hypertensive drug delivery in the spontaneously hypertensive rat model

**DOI:** 10.1371/journal.pone.0231244

**Published:** 2020-04-16

**Authors:** Hua Guo, Yuqing Wang, Wei Cai, Chengqi He

**Affiliations:** 1 Province Key Laboratory of Rehabilitation Medicine, Department of Rehabilitation Medicine, West China Hospital of Sichuan University, Chengdu, China; 2 Nanotechnology Development Department, National Center for Nanoscience and Technology of China, Beijing, China; 3 Department of Radiology, Beijing Jishuitan Hospital, 4th Clinical Medical College of Peking University, Beijing, China; Institute of Materials Science, GERMANY

## Abstract

**Background:**

Quantifying dose-dependent ultra-early edema and ultrastructural changes in the myocyte after drug delivery is important for the development of new mixed calcium channel blockers (CCBs).

**Materials and methods:**

Arterial cannulation was used to measure mean arterial pressure in real time; simultaneously, magnetic resonance imaging proton density mapping was used to quantify edema 5–55 min after the delivery of L-type CCBs, T- and L-type CCBs, and solvent to a spontaneously hypertensive rat model. Transmission electron microscopy was used to show ultrastructural changes in the myocyte.

**Results:**

Analysis of variance showed significant differences among the three groups in mean arterial pressure reduction (F = 246.36, P = 5.75E^-25^), ultra-early level of edema (ULE) (F = 175.49, P = 5.62E^-22^), and dose-dependent level of edema (DLE) (F = 199.48, P = 4.28E^-23^). Compared with the solvent’s mean arterial pressure reduction (2.65±6.56±1.64), ULE (1.16±0.09±0.02), and DLE (0.0010±0.0001±0.0000), post hoc tests showed that T- and L-type CCBs had better mean arterial pressure reduction (90.67±11.58±2.90, P = 1.06E^-24^ vs. 68.34±15.19±3.80, P = 1.76E^-12^), lower ULE (1.53±0.14±0.04, P = 4.74E^-9^ vs. 2.08±0.18±0.04, P = 2.68E^-22^), and lower DLE (0.0025±0.0004±0.0001, P = 1.14E^-11^ vs. 0.0047±0.0008±0.0002, P = 2.10E^-11^) than L- type CCBs. Transmission electron microscopy showed that T- and L-type CCBs caused fewer ultrastructural changes in the myocytes after drug delivery than L-type CCBs.

**Conclusion:**

T- and L-type CCBs produced less ultra-early and dose-dependent edema, fewer ultrastructural changes in the myocyte, and a greater antihypertensive effect. Proton density mapping combined with arterial cannulation and transmission electron microscopy allowed for quantification of ultra-early and dose-dependent edema, antihypertensive efficacy, and ultrastructural changes in the myocyte. This is important for the evaluation of induced vasodilatory edema.

## Introduction

Traditional CCBs exert their antihypertensive effect by selectively inhibiting the L-type Ca^2+^ channel (or dihydropyridine channel), thus dilating arteries through the blockage of calcium influx by binding to the A1 subunit in arterial smooth muscle cells (SMCs) and decreasing the cells’ excitability [1,2]. Conventional L-type calcium channel blockers (L-CCB), which are widely used for clinical antihypertensive treatment (based on their affinity for the blood vessels versus the heart muscle), selectively block those L-type Ca^2+^ channels that are primarily distributed in peripheral arterioles. The antihypertensive effect is enhanced when L-CCBs are combined with other drugs such as angiotensin II receptor antagonists [3]. L-CCBs have a powerful antihypertensive effect and are generally well-tolerated and safe, but some adverse effects are commonly seen, including flushing, headache, dizziness, and vasodilatory edema, for which the incidence is 17% higher with L-CCBs compared with other CCBs [4]. This effect on vasodilatory edema is thought to be secondary to a disproportionate distribution of L-type Ca^2+^ channels, which results in increased hydrostatic pressure in the capillary circulation and the movement of fluid into the interstitial compartment [5].

Therefore, the correct combination of various CCB subtypes, which could block different Ca^2+^ channels that are distributed in both the peripheral arterioles and venules, could simultaneously improve the antihypertensive effect and alleviate vasodilatory edema [6]. Notably, substantial differences in blood pressure responses among ethnic groups to first- and second-line antihypertensive drugs have been found, introducing another factor that may influence vasodilatory edema [7]. One study showed that T-type Ca^2+^ channels play a pivotal role in the regulation of afferent and efferent arterioles, and in the mediation of Ca^2+^ influx that is related to angiotensin-induced afferent and efferent arteriolar vasoconstriction [8]. Mibefradil is an example of a T- and L-type CCB (T&L-CCB) that has this effect. It selectively blocks T-type calcium channels, unlike other types of calcium channel antagonists that block only L-type channels [5,9,10].

Although the relationship between vasodilatory edema and the mechanism of action of CCBs is relevant, ultra-early and dose-dependent edema, antihypertensive efficacy, and ultrastructural changes of the myocyte after drug delivery are factors that are more important to research.

For example, ultra-early lesions have proven to be sensitive to the proton density mapping (PD-mapping) method, which is based on magnetic resonance imaging (MRI) [11]. The PD-mapping method has been used to measure ultra-early edema in recent studies, including the present study. Specifically, increased T2 signal intensity is secondary to the osmotic shift of muscle water, which leads to an increase in the intracellular space [12]. Another important method of structural evaluation is transmission electron microscopy (TEM), which operates on the same basic principles as light microscopy but with the use of electrons instead of light. TEM has been used widely to describe the ultrastructure of the myocyte [13, 14].

In summary, these methods provide support for the evaluation of vasodilatory edema and ultrastructural changes in the myocyte in general, and specifically at the ultra-early stage and over the entire duration of drug delivery. Discovering whether there are differences in the evolution of edema that depend on the CCB dose and on how early the edema occurs is important for describing the drugs’ effect on hypertension. Therefore, to quantify the antihypertensive effects produced by L- and T&L-CCB delivery in this study, arterial cannulation was used to measure (in real time) the mean arterial pressure (MAP) and to evaluate the antihypertensive effects of CCBs. Simultaneously, the PD-mapping method was used to quantify ultra-early and dose-dependent vasodilatory edema evoked by L- and T&L-CCBs delivery. Given the utility of the available imaging modalities, TEM was used to describe the ultrastructural changes in the myocyte, and when combined with MRI, TEM was used to evaluate the vasodilatory edema induced by equivalent doses of T&L-CCB and L-CCB. We aimed to accurately quantify the ultra-early and dose-dependent differences in edema and describe the ultrastructural changes in the myocyte between L- and T&L-CCB after drug delivery.

## Materials and methods

### Materials

All animal protocols were approved by the Institutional Animal Ethical and Welfare of West China Hospital of Sichuan University

Two types of CCBs were evaluated in the present study. Both Nifedipine (L-CCB) and Mibefradil (T&L-CCB) were obtained from Sigma-Aldrich (St. Louis, MO).

### Animal preparation

The animal handling and imaging procedures, based on the Institute of Laboratory Animal Resources, were approved by the local institutional animal care and use committee.

Forty-eight spontaneously hypertensive rats (SHRs), aged 17 weeks, were included based on their middle blood pressure (MBP > 150 mmHg) and were divided randomly into three groups and fed adaptively for one week. Each SHR was strapped on the operation table after intraperitoneal injection with pentobarbital sodium. Based on a previous study, left carotid artery cannulation was performed to measure MAP in real time [5]. The right jugular vein cannula was connected to a microsyringe pump for drug delivery (Table 1). Subsequently, each SHR was scanned with inhalational anesthesia and maintained at a temperature of 36 ± 1°C by a thermostatic water tank.

**Table 1:** Groups of experimental animals and drugs.

Group	Animal	Number	Dosage (mg/kg)
Solvent	SHR	16	/
L-CCBs	SHR	16	1
T&L-CCBs	SHR	16	10

### MRI acquisition

The MR images were acquired on a 7-Tesla Bruker BioSpec 70/20 USR MRI system (Bruker, BioSpin, Ettlingen, Germany) using a rat-body volume coil. A multi-slice multi-echo spin echo (MSME) sequence was used to obtain axial PD maps of the thigh. The scanning parameters were listed as follows: matrix = 128 × 128; field-of-view (FOV) = 50 × 50 mm^2^; number of slices = 18; slice thickness = 1 mm; repetition time (TR)/echo time (TE) = 2500/6.5 ms; and echo train length = 12. The acquisition time of each MSME PD mapping sequence was approximately 5 min.

### Drug delivery

Each SHR was scanned dynamically using the PD mapping sequence before and after drug delivery at multiple time points (6 times from 5 to 55 min). Based on the previous study, half the dose was injected initially, and the other half was subsequently injected by a microsyringe pump over 55 min [5]. During PD-mapping scans, the average MAP value for each SHR was recorded simultaneously in real time using an electrophysiological instrument (Pclab-3804, Beijing, MICROSIGNALSTAR Technology).

### MRI data analysis

Regions of interest (ROIs) were drawn on nine contiguous slices of the bilateral thigh muscles with the bone structures excluded. Within each ROI, pixel-by-pixel PD values were calculated using negative exponential curve fitting of the signal intensities (SI) of T2-weighted images acquired at different TEs (Eq 1) [11,12].

$$SI=K\times PD\times e^{\frac{TE}{T2}}\times (1-e^{\frac{TR}{T1}})$$ (1)

where K is a constant, and $1-e^{\frac{TR}{T1}}$ is a fixed term at each TE that was cancelled out in the exponential fitting.

To evaluate the edema after drug delivery, PD values measured at each time point (t) relative to PD acquired immediately before drug delivery *PD*(0) were calculated as follows (Eq 2):

ΔPD(t)=(PD(t)−PD(0))/PD(0) (2)

Linear regression using the Levenberg-Marquardt method was performed on the time series of PD measurements to derive the relationship between PD and t (Eq 3):

ΔPD(t)=a×t+b (3)

Two indexes, the ultra-early level of edema (ULE) and the dose-dependent level of edema (DLE), were calculated to quantify the ultra-early and dose-dependent edema after drug delivery, respectively.

$$ULE=\int_{0}^{t}\Delta PD(\tau )d\tau ,t=5\min$$ (4)

$$DLE=\frac{d\Delta PD(t)}{dt}$$ (5)

The data analyses were developed using Matlab software (The MathWorks Inc., Natick, Massachusetts).

### TEM analysis

After MRI scanning, the SHRs were sacrificed by intravenous injection with pentobarbital sodium, and tissue samples of 1 × 1 × 2 mm dimension were extracted from the quadriceps femoris for electron microscope observation. The isolated tissues were fixed and preserved in 3% glutaraldehyde solution during the preparation of the specimens. The specimens were observed using TEM (HT-7700, Japan, Hitachi) to evaluate the alternations of myocyte ultrastructure induced by vasodilatory edema.

### Statistical analysis

Continuous variables were expressed as the mean±SD±SEM. Comparisons among different drug treatment groups were analyzed by using the one-way ANOVA method. Post hoc tests were performed using the Bonferroni or Tamhane method adjustment according to the homogeneity of variance. All the analyses were performed using SPSS17. A p-value less than 0.05 were considered statistically significant.

## Results

### MAP reduction

**Fig 1:**
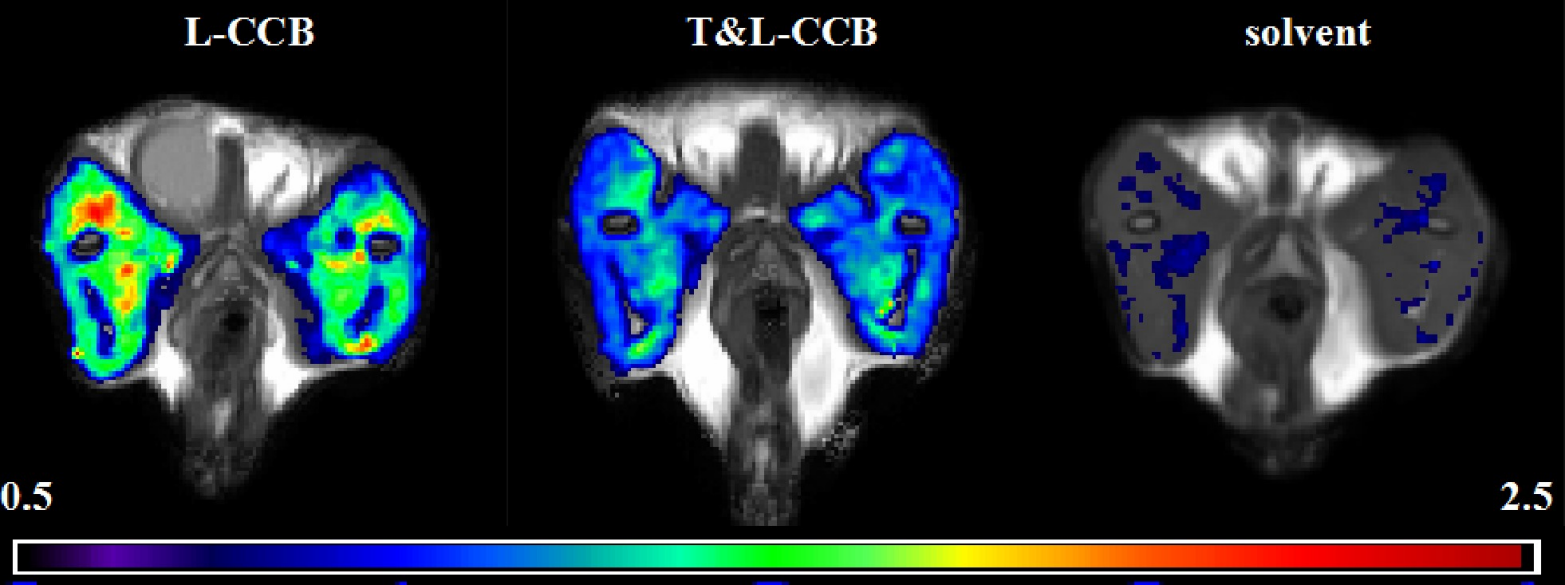
The antihypertensive effects of each drug and the solvent.

There were significant differences among the three groups of MAP reductions (F = 246.36, P = 5.75E^-25^ <0.001), and the Tamhane method was applied to the post hoc tests in terms of the homogeneity test of variance (Leven statistic = 8.74, P = 6.19E^-4^ <0.001). In Fig 1, the drugs demonstrated better antihypertensive effects than the solvent (P<0.001), as shown in Table 2. Compared with the solvent (2.65±6.56±1.64), the T&L-CCB showed better MAP reduction (90.67±11.58±2.90, P = 1.06E^-24^) than L-CCB (68.34±15.19±3.80, P = 1.76E^-12^).

**Table 2:** Details of MAP reductions and edema for each drug delivery group.

Drugs	MAP reduction (mmHg) Mean SD SEM	ULE Mean SD SEM	DLE Mean SD SEM
Solvent	2.65 6.56 1.64	1.16 0.09 0.02	0.0010 0.0001 0.0000
L-CCBs	68.34 15.19 3.80	2.08 0.18 0.04	0.0047 0.0008 0.0002
T&L-CCBs	90.67 11.58 2.90	1.53 0.14 0.04	0.0025 0.0004 0.0001

### ULE

There were significant differences among the three ULE groups (F = 175.49, P = 5.62E^-22^<0.001), and the Bonferroni method was applied to the post hoc tests in terms of the homogeneity test of variance (Leven statistic = 1.89, P = 0.163), as shown in Table 1. Compared with the solvent (1.16±0.09±0.02), the T&L-CCB revealed a lower ULE (1.53±0.14±0.04, P = 4.74E^-9^) than L-CCB (2.08±0.18±0.04, P = 2.68E^-22^), as shown in Table 1.

**Fig 2:**
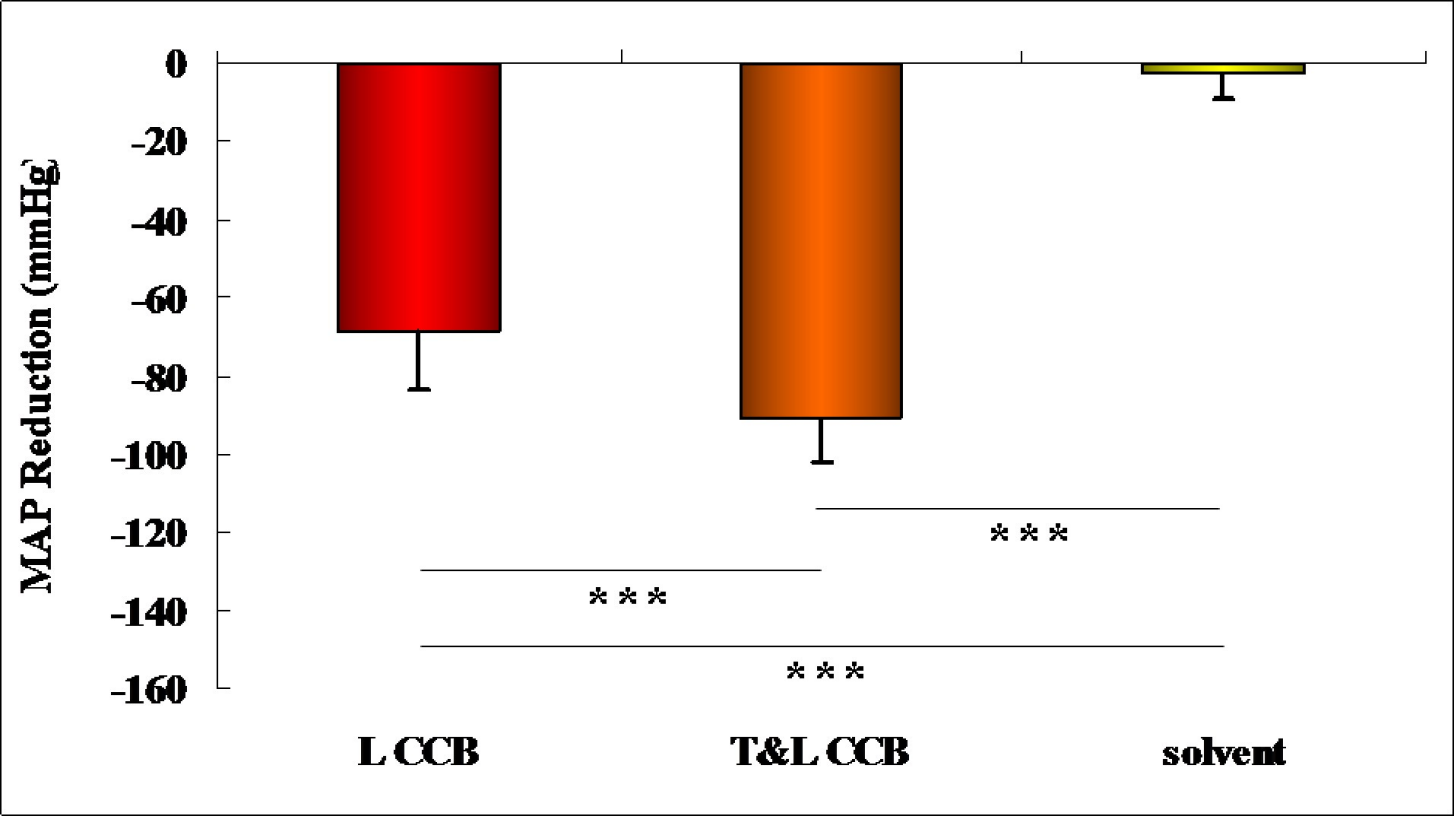
ULE map after drug delivery.

**Fig 3:**
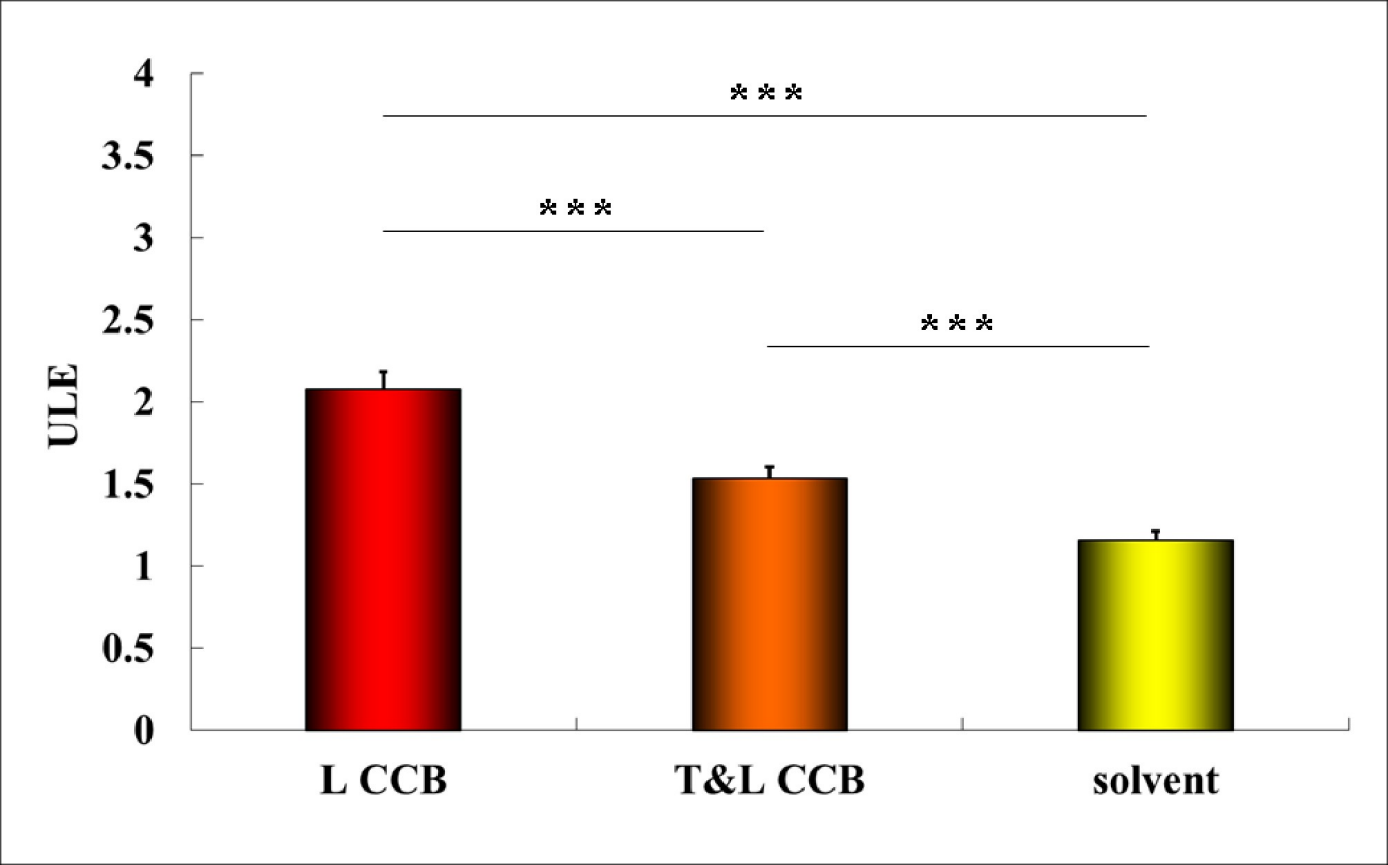
ULE of each drug and the solvent.

The ULE maps were overlaid on PD-weighted images of the bilateral thighs. In Fig 2, compared with the solvent, T&L-CCB showed lower ULE values, but L-CCB revealed higher ULE values. In Fig 3, both L-CCB and T&L-CCB revealed significantly increased ULE (P<0.001).

### DLE

**Fig 4:**
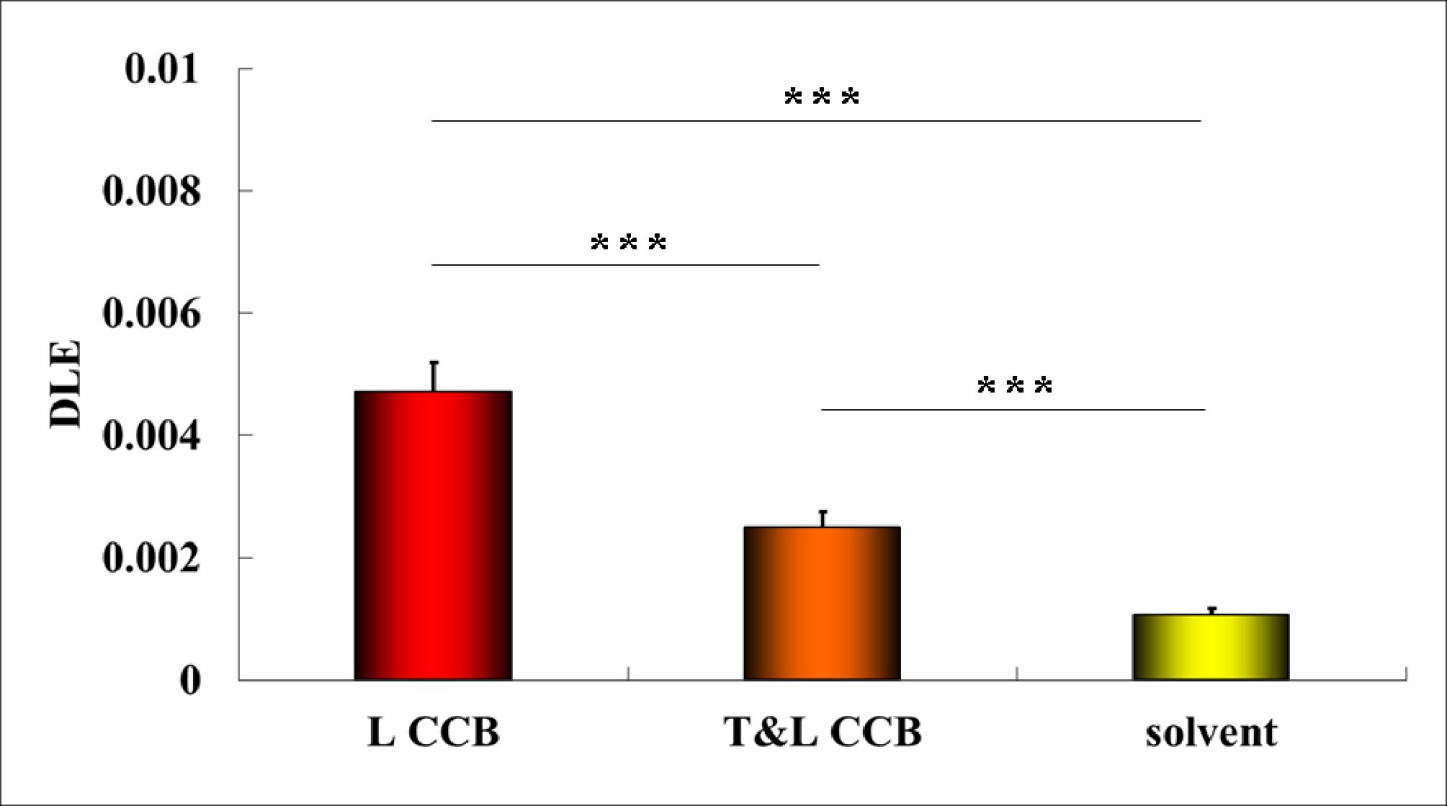
DLE of each drug and the solvent.

There were significant differences among the three DLE groups (F = 199.48, P = 4.28E^-23^<0.001), and the Tamhane method was used for the post hoc tests in terms of the homogeneity test of variance (Leven statistic = 12.08, P = 6.32E^-5^<0.001) (Table 2). In Fig 4, all of the drugs showed significantly increased DLE (P<0.001). Compared with the solvent (0.0010±0.0001±0.0000), the T&L-CCB showed a lower DLE (0.0025±0.0004±0.0001, P = 1.14E^-11^) than L-CCB (0.0047±0.0008±0.0002, P = 2.10E^-11^).

### Myocyte microstructures

**Fig 5:**
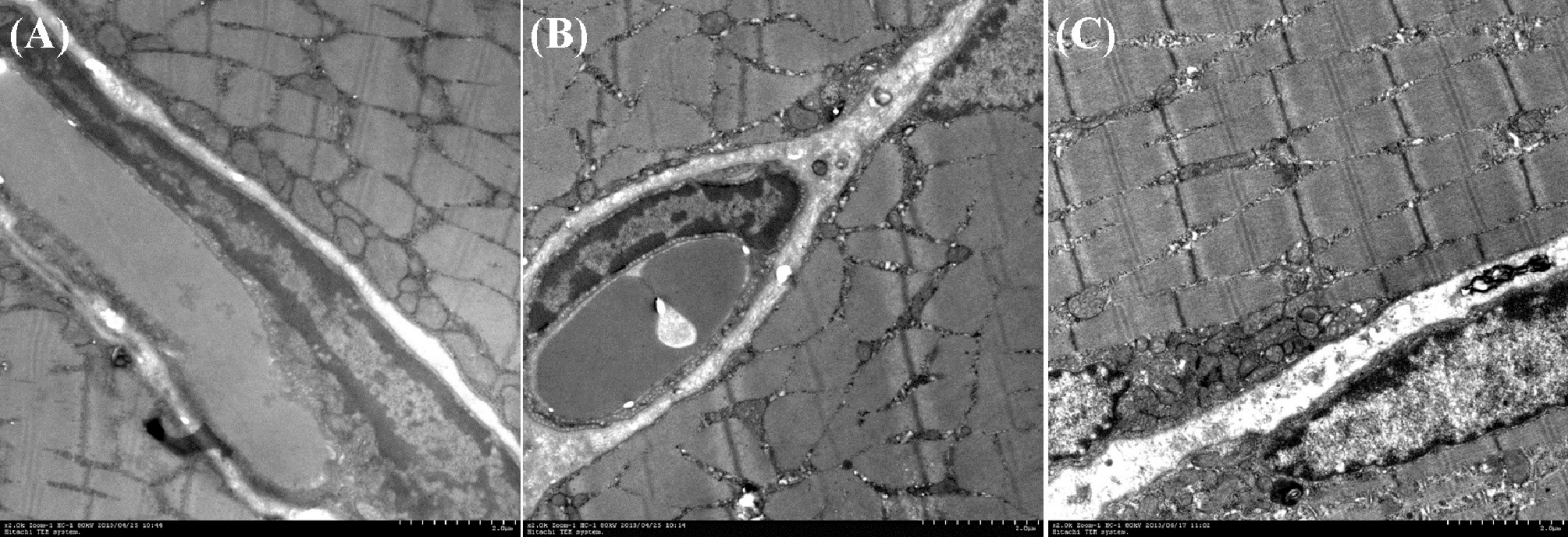
TEM of myocyte structure for each drug delivery. (A) L-CCB group. (B) T&L-CCB group. (C) Solvent group.

In the L-CCB group, the damage in the blurry light and dark bands of the sarcomere was severe, the sarcoplasmic reticulum had large lipid vesicles, and the cell nucleus was extruded and deformed (Fig 5). (A) The L-CCB group showed ruptured, blurry, light, and dark sarcomere bands, large lipid vesicles in the sarcoplasmic reticulum, and an extruded and deformed cell nucleus. (B) The T&L-CCB group showed deformed, ruptured, and blurry light and dark bands, swollen cells in the sarcoplasmic reticulum, vesicle formation, and a deformed cell nucleus. (C) The solvent group showed light and dark sarcomere bands, visible mitochondria, intact cells in the sarcoplasmic reticulum, and many mitochondria and endoplasmic reticulum in the cytoplasm. In T&L-CCB group, light and dark bands were deformed, ruptured, and blurry, cells in the sarcoplasmic reticulum were swollen and demonstrated vesicle formation, and the cell nucleus were deformed. In the solvent group, light and dark sarcomere bands were present, mitochondria were visible, cells in the sarcoplasmic reticulum were intact, and the cytoplasm had plentiful mitochondria and endoplasmic reticulum.

## Discussion

Compared with L-CCB, T&L-CCB had a better antihypertensive effect, slightly less ultra-early and dose-dependent edema, and caused milder ultrastructural changes in the myocytes, making it an effective choice for the development of a new antihypertensive drug. The benefits of a combination of more than one antihypertensive drug over monotherapy has been demonstrated in the literature [15,16]. The MRI PD-mapping, combined with the arterial cannula and TEM, provided a precise solution to evaluate the antihypertensive effect of newly developed drug, as well as to assess ultra-early and dose-dependent edema, and ultrastructural changes in the myocyte after drug delivery, all of which are important for exploring the mechanism of induced vasodilatory edema. Moreover, the findings of this study also suggested that MRI is an accurate and sensitive method for quantitatively evaluating the preclinical antihypertensive effect and adverse effects, which would be of great value in translational medicine.

### The potential mechanism of T- or T&L- CCBs

Like first-line antihypertensive drugs, the potent CCB vasodilators do not affect venous tone [17]. Although the expression levels of L-type Ca^2+^ channels in small arteries and their venous counterparts are similar, their function is inactivated by Ca^2+^ from intracellular stores in the SMCs of small veins [6]. This preferential inhibition leads to the extravasation of fluids and also produces peripheral edema, which is the most common adverse effect of L-CCBs [2]. In an attempt to avoid this adverse effect, CCBs that block dual-type or other Ca^2+^ channels (e.g., T- or T&L-CCB) have been evaluated in some studies [5,9]. These studies confirm that T- or T&L-CCB only evoke milder interstitial edema after drug delivery with equal doses. The reason is that the T-type Ca^2+^ channels are indistinguishably expressed in small arteries and veins that avoid significant increments of capillary osmotic pressure. Our results confirmed that interstitial edema by T&L-CCB was significantly alleviated compared with L-CCB.

### The physiological significance of PD values for vasodilatory edema

The PD value is reflective of the abundance of free water in the biological tissues [11]. After drug delivery, the hydrostatic pressure in the capillary circulation is increased due to the disproportionate expression of Ca^2+^ channels in small arteries and veins [5]. Subsequently, more free water permeates into the interstitial compartment, producing vasodilatory edema. This edema can be measured by incremental PD values in the tissues after drug delivery. Moreover, the dynamic changes of PD values during the process of drug delivery can be used to quantify the variation tendency of vasodilatory edema induced by antihypertensive drugs. Our results confirmed that the PD values could be used to quantify the level and variation tendency of vasodilatory edema at different stages, both at the ultra-early stage and during the entire drug delivery period.

### Ultra-early vasodilatory edema

After drug delivery with L-CCB or T&L-CCB, little difference in the extent of vasodilatory edema was seen because the vasodilatory edema evoked by disproportionate dilation between the arteriole and venule was milder at the ultra-early stage. This integral method could aggregate fewer increments of PD values 5 min after drug delivery, which would enhance the difference in performance between two drugs. Our results confirmed that the integral method could identify the lack of differences at the ultra-early stage of edema. Therefore, we propose that this method is suitable for quantifying ultra-early edema produced by new drugs. In our study, the L-CCB group showed higher ULE values (i.e., greater initial edema), but the T&L-CCB group only showed lower ULE values (i.e., less initial edema). Therefore, this method is important in the development of new drugs to evaluate the efficacy and adverse effects at the ultra-early stage because the initial physiological changes would occur then.

### Dose-dependent vasodilatory edema

In our study, the strategy of drug delivery was to inject half the dose initially, followed by injection of the other half of the dose by microsyringe pump for 55 min. Our hypothesis was that less vasodilatory edema would occur at the early stage after drug delivery, and that the level of edema would increase over time. A previous study confirmed that the level of vasodilatory edema is proportional to the drug dose [17]. Therefore, we proposed that the first half-dose only produced the initial edema at the early stage and the other half-dose produced successive increments of edema over the entire duration of drug delivery. In our study, the initial edema was produced at the ultra-early stage and the level of edema subsequently increased gradually over time, consistent with our hypothesis. Based on the consistency between our results and this hypothesis, we believe that our method is suitable to quantify the efficacy and adverse effects of the new drug *in vivo*. In our study, the L-CCB group achieved higher DLE values (i.e., higher dose-dependent increments in edema), but the T&L-CCB group only showed lower DLE values (i.e., lower dose-dependent increments in edema). Therefore, this method was also suitable for optimizing the drug dose.

### Ultrastructural changes of vasodilatory edema

After drug delivery, more free water permeates into the intercellular space due to the unbalanced hydrostatic pressure in the capillary circulation [5]. The excess aggregation of free water in the intercellular space enlarges the space between the histocyte and capillary circulations, which delays the transportation of oxygen and nutrient substances to the histocytes [18]. More intercellular fluids impacts the local capillary circulation and reduces the blood supply to histocytes, which results in dystrophia and decreased energy metabolism in the cells. Therefore, we proposed that the myocyte ultrastructure would suffer varying degrees of damage over time after drug delivery, and the degree of damage was proportional to the level of edema. Based on the results of the PD map, we found that the ULE and DLE values in the L-CCB group were higher than those in the T&L-CCB group, which produced a higher initial level of edema and dynamic increments in edema in the L-CCB group. The result of TEM also revealed a mass of vesicles in the sarcomere, mitochondria, and endoplasmic reticulum, swollen organelles, and increased partial vesicle formation, leading to more severe ultrastructural damage in the L-CCB group. However, a small number of lipid-like vesicles in the sarcomere produced less ultrastructural damage in the T&L-CCB group. Our study findings were confirmed by the consistency between MRI and TEM. Moreover, our results suggested that dynamic PD-mapping combined with arterial cannulation and TEM provide a precise solution for newly developed drugs, which was important for quantification of the efficacy and adverse effects, in addition to exploration of the mechanism of induced vasodilatory edema.

This study had several limitations. There were too few TEM results to describe the dynamic ultrastructural changes of vasodilatory edema 5–55 min after drug delivery. These results were important to show the invasive process of edema from the extracellular space to the myocyte. Further research work will be conducted in the future to better understand the mechanism of vasodilatory edema.

## Conclusions

In summary, this study indicated that the T&L-CCBs are an effective choice for the development of new antihypertensive drugs. PD-mapping combined with arterial cannulation and TEM provided a precise solution for newly developed drugs to effectively evaluate ultra-early and dose-dependent edema, antihypertensive efficacy, and ultrastructural changes in the myocyte after drug delivery. This is important for exploration of the induced mechanism of vasodilatory edema. Moreover, the findings of this study suggested that MRI is an accurate, sensitive and convenient measurement tool [19] that can be used to quantitatively evaluate preclinical antihypertensive effects and adverse effects, and these factors are of great value in translational medicine.

## Supporting information

S1 Checklist A copy of the ARRIVE guidelines checklist. (DOC)

S1 Data (RAR)

S1 File (RAR)

## References

[1] Huber I, Wappl E, Herzog A, Mitterdorfer J, Glossmann H, Langer T et al Conserved Ca^2+^-antagonist-binding properties and putative folding structure of a recombinant high-affinity dihydropyridine-binding domain. Biochem J. 2000;347 Pt 3:829–36. 10769189 PMC1221022

[2] Safak C, Simsek R. Fused 1,4-dihydropyridines as potential calcium modulatory compounds. Mini Rev Med Chem. 2006 7;6(7):747–755 10.2174/138955706777698606 16842124

[3] Karpov Y, Dongre N, Vigdorchik A, Sastravaha K. Amlodipine/valsartan single-pill combination: a prospective, observational evaluation of the real-life safety and effectiveness in the routine treatment of hypertension. Adv Ther. 2012 2 29(2):134–47. 10.1007/s12325-011-0095-0 22271158

[4] Kobrin I, Charlon V, Bursztyn M. New developments in drug therapy of hypertension. Med Clin North Am.1997 11;81(6):1359–71. 10.1016/s0025-7125(05)70588-5 9356604

[5] Major TC, Dhamija S, Black N, Liachenko S, Morenko B, Sobocinski G et al The T- and L-type calcium channel blocker (CCB) mibefradil attenuates leg edema induced by the L-type CCB nifedipine in the spontaneously hypertensive rat: a novel differentiating assay. J Pharmacol Exp Ther. 2008 6;325(3):723–31. 10.1124/jpet.107.133892 18326812

[6] Feng MG, Navar LG. Angiotensin II-mediated constriction of afferent and efferent arterioles involves T-type Ca2+ channel activation. Am J Nephrol. 2004 Nov-Dec;24(6):641–8. Epub 2004 Dec 23. 10.1159/000082946 15627720

[7] Gupta AK, Poulter NR, Dobson J, Eldridge S, Cappuccio FP, Caulfield M. Ethnic differences in blood pressure response to first and second-line antihypertensive therapies in patients randomized in the ASCOT Trial. Am J Hypertens. 2010 9;23(9):1023–30. 10.1038/ajh.2010.105 20725056

[8] Ozawa Y, Hayashi K, Nagahama T, Fujiwara K, Saruta T. Effect of T-type selective calcium antagonist on renal microcirculation: studies in the isolated perfused hydronephrotic kidney. Hypertension. 2001 9;38(3):343–7. 10.1161/01.hyp.38.3.343 11566902

[9] Brogden RN, Markham A. Mibefradil. A review of its pharmacodynamic and pharmacokinetic properties, and therapeutic efficacy in the management of hypertension and angina pectoris. Drugs. 1997 11;54(5):774–93. Erratum in: Drugs 1998 Apr;55(4):517. 10.2165/00003495-199754050-00010 9360062

[10] Levine TB, Bernink PJ, Caspi A, Elkayam U, Geltman EM, Greenberg B et al Effect of mibefradil, a T-type calcium channel blocker, on morbidity and mortality in moderate to severe congestive heart failure: The MACH-1 study. Mortality assessment in congestive heart failure trial. Circulation. 2000 2 22;101(7):758–764. 10.1161/01.cir.101.7.758 10683349

[11] Klengel A, Stumpp P, Klengel S, Bottger I, Ronisch N, Kahn T. Detection of Traumatic Bone Marrow Lesions after Knee Trauma: Comparison of ADC Maps Derived from Diffusion-weighted Imaging with Standard Fat-saturated Proton Density-weighted Turbo Spin-Echo Sequences. Radiology. 2016 5;283(2):467–477. 10.1148/radiol.2016160306 27775896

[12] Tawara N, Nitta O, Kuruma H, Niitsu M, Itoh A. T2 mapping of muscle activity using ultrafast imaging. Magn Reson Med Sci. 2011;10(2):85–91. 10.2463/mrms.10.85 21720110

[13] Patten C, Meyer RA, Fleckenstein JL. T2 mapping of muscle. Semin Musculoskelet Radiol. 2003 12;7(4):297–305. 10.1055/s-2004-815677 14735428

[14] Zhao J, Song Q, Wang L, Dong X, Yang X, Bai X et al Detrusor myocyte autophagy protects the bladder function via inhibiting the inflammation in cyclophosphamide-induced cystitis in rats. PLoS One. 2015 4 1;10(4):e0122597 10.1371/journal.pone.0122597 25830308 PMC4382282

[15] Messerli FH. Vasodilatory edema: a common side effect of antihypertensive therapy. Am J Hypertens. 2001 9;14(9 Pt 1):978–9. 10.1016/s0895-7061(01)02178-1 11587169

[16] Weir MR, Rosenberger C, Fink JC. Pilot study to evaluate a water displacement technique to compare effects of diuretics and ACE inhibitors to alleviate lower extremity edema due to dihydropyridine calcium antagonists. Am J Hypertens. 2001 9;14(9 Pt 1):963–8. 10.1016/s0895-7061(01)02167-7 11587165

[17] Esposito A, Campana L, Palmisano A, De Cobelli F, Canu T, Santarella F. Magnetic resonance imaging at 7T reveals common events in age-related sarcopenia and in the homeostatic response to muscle sterile injury. PLoS One. 2013;8(3):e59308 10.1371/journal.pone.0059308 23555016 PMC3595251

[18] Messerli FH. Vasodilatory edema: a common side effect of antihypertensive therapy. Curr Cardiol Rep. 2002 11;4(6):479–482. 10.1007/s11886-002-0110-9 12379167

[19] Zhang LY, Ding JT, Wang Y, Zhang WG, Deng XJ, Chen JH. MRI quantitative study and pathologic analysis of crush injury in rabbit hind limb muscles. Journal Surg Research. 2011 5 15;167(2):e357–363 21035134 10.1016/j.jss.2010.09.014

