# Supplementary figures and images for: Evaluation of ultra-early and dose-dependent edema and ultrastructural changes in the myocyte during anti-hypertensive drug delivery in the spontaneously hypertensive rat model
Source: PLoS One. 2020 Apr 16;15(4):e0231244. doi: 10.1371/journal.pone.0231244 (PMC7162487; doi:10.1371/journal.pone.0231244)

**1.** **i） A copy of the ARRIVE Guidelines checklist**

**
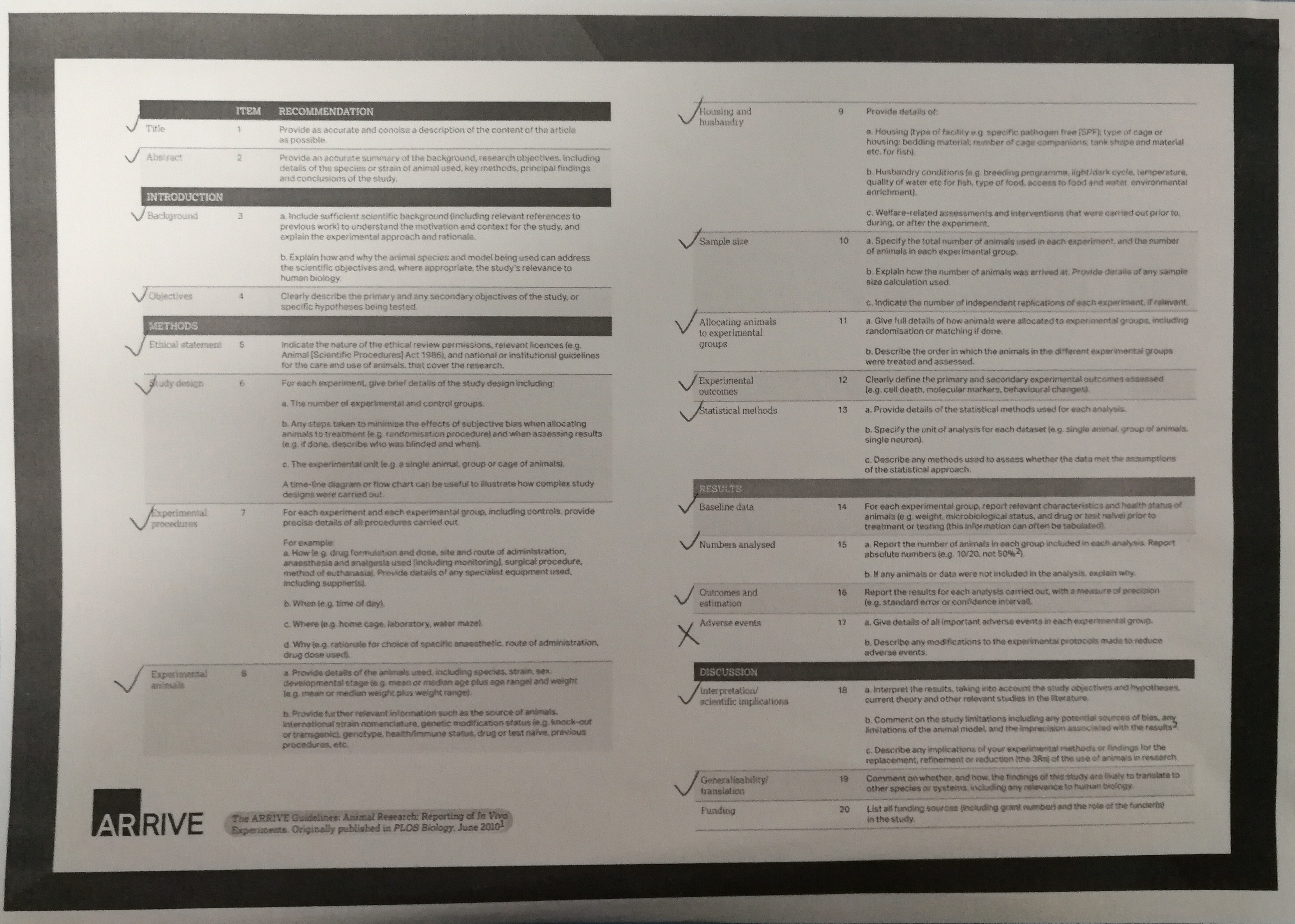
**

Supplement: S1 Checklist — (DOC) [file pone.0231244.s001.doc]
